# Online academic satisfaction during the COVID-19 pandemic in medical students: role of sleep, emotions, college adjustment, and digital skills

**DOI:** 10.12688/f1000research.76127.1

**Published:** 2022-02-28

**Authors:** Sebastian A. Medina-Ramirez, Ricardo Rojas-Humpire, Josue F. Canaza, Fiorella Hernandez, Salomón Huancahuire-Vega

**Affiliations:** 1 Department of Basic Sciences, Peruvian Union University (UPeU), Lima, Lima, 15, Peru; 2 Department of Basic Sciences, Human Medicine School, Peruvian Union University (UPeU), Lima, Lima, 15, Peru; 3 P53 Research Group, Peruvian Union University (UPeU), Lima, Lima, 15, Peru; 4 P53 Research Group, Human Medicine School, Peruvian Union University (UPeU), Lima, Lima, 15, Peru; 5 Research General Directorate, Peruvian Union University (UPeU), Lima, Lima, 15, Peru

**Keywords:** COVID-19, Medical students, academic satisfaction, sleep, digital competencies

## Abstract

**Background:** The measures taken to contain the COVID-19 pandemic, led to significant changes in university education, resulting in the new normal standard of virtual teaching in many undergraduate medical schools worldwide. Therefore, the aim of this paper was to determine the factors related to academic satisfaction with virtual teaching in medical students during the COVID-19 pandemic.

**Methods: **A cross-sectional-analytical study was conducted on medical students at a private university in Peru, through self-reported questionnaires divided into sociodemographic data and variables of interest that could influence academic satisfaction during the pandemic. To evaluate possible factors related to academic satisfaction, stepwise regression models were performed for both sexes.

**Results:** In total, data from 310 medical students, 117 males and 193 females, were analyzed. Academic satisfaction reached a score of 11.2 ± 2.9, which was similar in both sexes. The best regression model for males (AIC: 544.32; RMSE: 2.42; R ^2^: 0.30) showed that adaptation to university life (favorable change) and depression (unfavorable change) explained 30% of changes in students' academic satisfaction. While in females (AIC: 907.59; RMSE: 2.49; R ^2^: 0.22) the model integrated favorable factors such as adjustment to college life and anxiety; while depression and poor sleep quality were unfavorable factors.

**Conclusion:** Factors that contributed to academic satisfaction in medical students were determined in this study, which differed by gender. Thus, it is important to take into account the particularities of male and female medical students in order to improve their academic satisfaction during their university careers.

## Introduction

On March 11, 2020, the World Health Organization declared the outbreak of coronavirus disease (COVID-19) a pandemic. Since then, strict measures have been taken to contain the spread of the virus. Social distancing being the main one, has brought with it significant problems for education as it affects nearly 1.6 billion students worldwide. ^1^ This posed a substantial challenge to medical education forcing an abrupt transition to online formats. ^2^ Due to restrictions, there were limitations of access the suitable educational environments (such as laboratories, simulation rooms, and hospitals) leading to rapid transition to remote learning. As a result, there may be skills and health factors that would be impacting students' individual experience and perception of their academic environment. ^3^ In addition, medical students, as well as other students in higher education, experience various difficulties that lead to a general feeling of dissatisfaction and frustration with online education. ^4^

Academic satisfaction in academic environments through a socio-cognitive model has been highlighted in several studies due to its importance in finding external factors that could have an impact on students' overall academic satisfaction. ^5^ In this model, cognitive, affective, and behavioral factors are considered due to students' perceptions of their performance contrary to other studies that focus on the subject's perception in the institutional context. ^6^ Due to the COVID 19 pandemic, medical students in Peru have had no choice but to transition to remote education and it is not currently known how satisfied they are with their studies.

In addition, it is important to know the influence that the pandemic has on mental health in college students, previous studies have shown that there is an association with depression and anxiety. ^7^ On the other hand, for the use of technology, the success of acquiring a good experience will depend on the learning obtained by using it, whether for academic or non-academic purposes. ^8^ In the context of the pandemic, this could influence the experience of using and acquiring digital skills required for optimal learning. Factors such as mental health issues and the need to adapt to the digital environment could be contributing to perceptions of academic satisfaction.

Understanding the factors that positively or negatively influence academic satisfaction could lead us to effectively assess the problem and design targeted interventions according to the needs of a specific group of individuals classified by gender, age or socio-demographic origin. In our study we included variables previously known to have some influence on students' academic satisfaction such as adaptability to university life, digital skills, sleep quality, anxiety and depression. The aim of this work was to determine the factors related to academic satisfaction in medical students during the COVID-19 pandemic.

## Methods

### Study design and population

This analytical cross-sectional study was conducted in students of the Faculty of Medicine of the Universidad Peruana Unión belonging to the first to the seventh year of studies. Students were invited to complete an online survey, in which questionnaires related to academic satisfaction, adaptation to the university, digital skills, sleep quality, stress, depression and anxiety were applied. Likewise, a virtual meeting was held for each academic year and a reminder to complete the survey was sent, which was available for 5 weeks. This study was reviewed and approved by the ethics committee of the Universidad Peruana Unión before being conducted (2021-CEUPeU-0005).

### Consent

Through a class briefing session per academic year, under the supervision of the academic area coordinator, all students were briefly informed of the purpose of the study and were given an average of 15 minutes to complete the questionnaire. Before completing the questionnaires, the participants who agreed to participate voluntarily gave their informed consent. Likewise, in the presentation, they were informed that the answers were confidential and that only the researchers had access to it for the purpose of this study.

### Measuring questionnaires

Socio-demographic aspects were considered in order to know the general information of the participants, and to measure the variables of interest we took into account five questionnaires, which are found in their completed version in extended data, and they are detailed below:

1.
*Brief Scale of Satisfaction with Studies (EBSE* *):* The EBSE is composed of 4 items, it is distributed in 2 factors: satisfaction with studies and negative affect. In this scale good internal consistency and reliability were evidenced (α=0.788). This scale reflects the satisfaction that the student has with respect to their performance and in general with their studies, distributed on a Likert scale of 5 options, ranging from strongly agree to strongly disagree. ^6^
2.
*Adaptability to university life* *:* Developed by Baker and Sirik, ^9^ and adapted by Rodriguez-Ayan and Sotelo. It is a questionnaire that aims to determine the degree of adaptation of students during their stay at the university. It measures 3 dimensions: social, academic and institutional. It consists of 11 items distributed on a 4-point Likert scale. ^10^
3.
*Pittsburgh Sleep Quality Index Questionnaire (PSQI* *):* PSQI is a questionnaire used to measure sleep quality and its alterations in the last month. ^11^ The questionnaire consists of 19 self-assessment questions that are used to obtain the overall score. Distributed in 7 items, which are: subjective sleep quality, latency, duration, efficiency, sleep disturbances, use of medication for sleep, and daytime dysfunction. In its original version this questionnaire has an internal consistency of (α=0.83), while the adapted version in the Peruvian population showed a lower consistency but was equally valid (α=0.564) and regarding construct validity, three factors were found that explain 60.2% of the total variance. ^12^
4.
*Digital skills* *:* This questionnaire consists of 35 questions measured using a 5-point Likert scale. It is based on the digital skills matrix developed by the National Autonomous University of Mexico (UNAM), exploring the following categories: access to information, communication and collaboration, information security, information management, media management, hardware and virtual learning environments. With respect to its psychometric characteristics, the study conducted by Avitia *et al.* recorded a good internal consistency (α=0.95) in general, as well as in each of its categories. ^13^
5.
*Depression, anxiety and stress scale (DASS - 21* *):* This questionnaire consists of 21 questions measured through a Likert scale from 0 to 3 points. It is divided into 3 dimensions which separately measures depression, stress and anxiety, each being composed of 7 items. In its Spanish version it has an acceptable internal validity, for stress (α=0.82), depression (α=0.84) and anxiety (α=0.70) and shows a good interrelation between the 3 factors evaluated by this scale. ^14^

### Data analysis

Data analysis was performed in the R programming language version 4.0.2. ( https://www.rstudio.com/) (RRID:SCR_001905). The variables were ordered in graphs and tables taking into account their categorical nature expressed as absolute frequency and percentage (%), or numerical as mean ± standard deviation (SD). For the comparative analysis, the χ ^2^ (chi-square) or Student's t-test was used according to the distribution of the variable. To establish the best model of factors related to academic satisfaction in medical students, stepwise regression models were performed with bidirectional elimination approach in the adjustment of independent variables, the best regression models were established based on Akaike's information criterion (AIC), root mean square error (RMSE), and R ^2^. Multivariate regression models stratified by sex were obtained with their respective 95% confidence intervals (95%CI), a value of p<0.05 was considered statistically significant in the analyses.

## Results

A total of 310 responses were obtained. The average age of the participants was 21.6 ± 3 years. The number of men was 117 (37.7%) and women 193 (62.3%). The total number of participants were divided into basic sciences (1 ^st^ and 2 ^nd^ academic year) and clinical sciences (3 ^rd^ to 7 ^th^ academic year), representing 42.3% and 57.7% respectively. It was found that half the participants belonged to the coastal region (52.9%), while those belonging to the highland region (21%) and those who were foreigners (20%) had similar proportion ( Table 1).

**Table 1.** General characteristics of medical students.

Variables	Total (n=310)	Men (n=117)	Women (n=193)	p-value
Age (years)	21.6 ± 3.0	21.6 ± 3.0	21.5 ± 2.9	0.63
Year of studies (%)				
First	66 (21.3)	27 (23.1)	39 (20.2)	0.983
Second	65 (21.0)	23 (19.7)	42 (21.8)	
Third	49 (15.8)	18 (15.4)	31 (16.1)	
Fourth	40 (12.9)	15 (12.8)	25 (13.0)	
Fifth	41 (13.2)	17 (14.5)	24 (12.4)	
Sixth	45 (14.5)	16 (13.7)	29 (15.0)	
Seventh	4 (1.3)	1 (0.9)	3 (1.6)	
Origin (%)				
Coast	164 (52.9)	64 (54.7)	100 (51.8)	0.531
Highlands	65 (21.0)	25 (21.4)	40 (20.7)	
Jungle	19 (6.1)	9 (7.7)	10 (5.2)	
Foreign	62 (20.0)	19 (16.2)	43 (22.3)	

Data expressed as mean ± SD or absolute frequency (%).

In the assessment of emotional disorders, the presence of at least one emotional disorder was evident in more than half of the respondents. However, this differed according to sex and type of emotional disorder.

**Figure 1.**
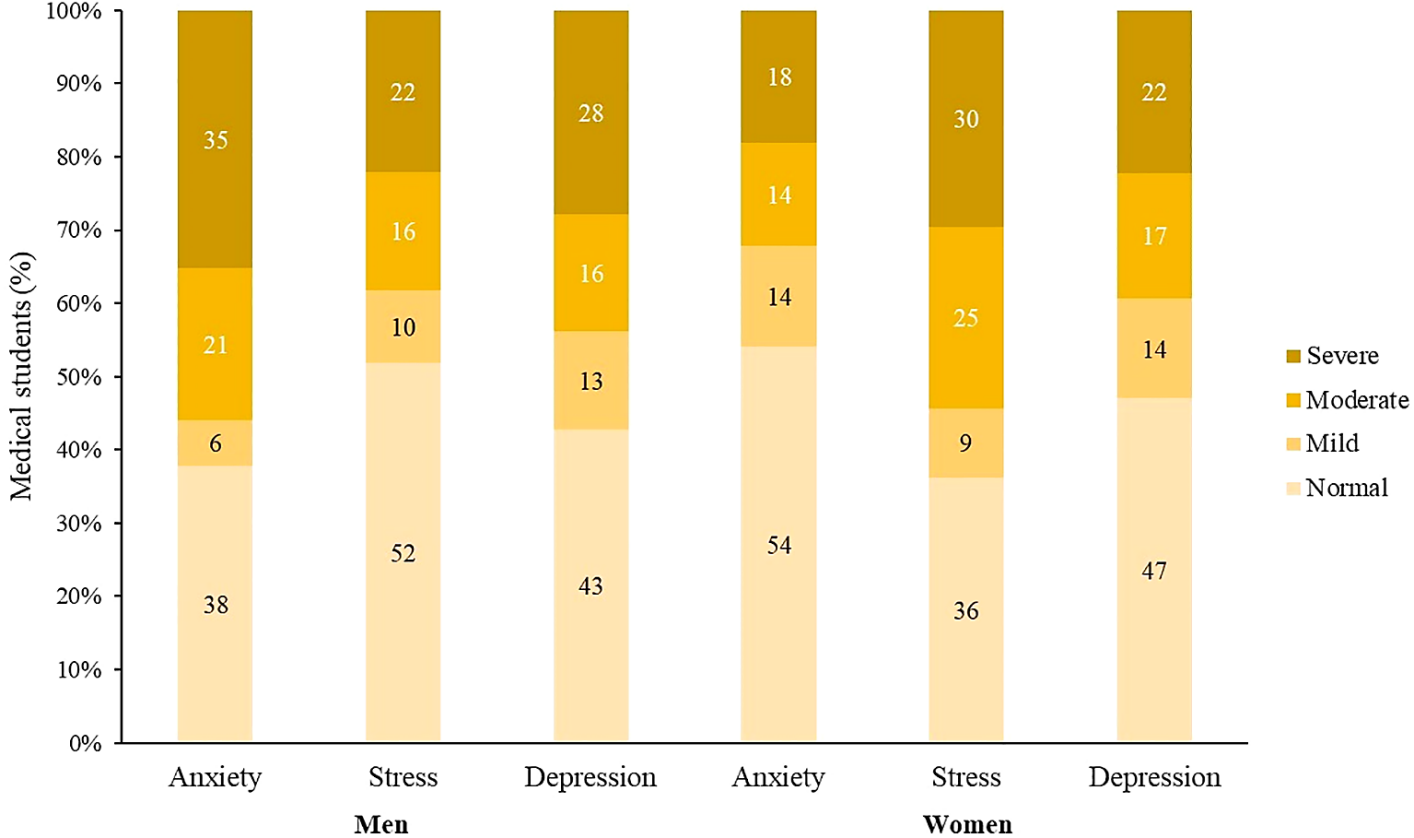
Distribution of emotional disorders in medical students.

In women, 64%, 53% and 46% had stress, depression and anxiety respectively, finding that 55% (n=106) of the stressors (moderate or severe) belonged to the most worrying states. However, in men, of the 62% who had anxiety, 56% (n=66) presented the most worrying states, while 57% and 48% had depression and stress respectively ( Figure 1).

A general result and comparison were made according to scores obtained in men and women for digital skills, adaptation to university life and academic satisfaction.

The results showed that, for digital skills, both sexes had a high score (87.5 ± 22.6, p=0.012), meaning a good management of digital programs or applications in general. Minimal differences in scores were found, interestingly it was statistically significant for women and men respectively in the following components: virtual environments (10 vs. 8.1, p<0.001), media management (7.7 vs. 6.9, p=0.021) and information management (15.6 vs. 14.2, p=0.007). This shows that there is a greater knowledge about the organization and use of the applications necessary for academic activities. However, this is contrary with respect to the management of search engines and access to online information, as well as a basic knowledge of information security, such as clearing history and logging out of the electronic devices they use.

On the other hand, for adaptation to university life we found an overall result of 36.9 ± 6.5 and for academic satisfaction of 11.2 ± 2.9, which did not present important differences in scores according to sex and were not statistically significant. Therefore, it is evident that they have the same perception in the new virtual teaching.

Regarding sleep quality, 83.9% (n=260) of the respondents had poor sleep quality, being the majority group in both sexes with this problem (163 women and 97 men), while only 16.1% (n=50) had good sleep quality ( Table 2).

**Table 2.** Digital skills, adaptation to university life and academic satisfaction of medical students.

Variables	Total (n=310)	Men (n=117)	Women (n=193)	p-value
Access to information ^a^	19.9 ± 5.7	19.4 ± 5.4	20.7 ± 6.0	0.082
Communication and collaboration ^a^	21.7 ± 6.1	21.5 ± 6.2	21.9 ± 5.9	0.586
Information Security ^a^	10.7 ± 3.3	10.5 ± 3.2	11.0 ± 3.4	0.170
Information Management ^a^	14.7 ± 5.1	14.2 ± 4.9	15.6 ± 5.3	0.007 *
Media Management ^a^	7.2 ± 2.8	6.9 ± 2.7	7.7 ± 2.9	0.021 *
Hardware ^a^	4.4 ± 1.9	4.4 ± 1.9	4.6 ± 1.9	0.235
Virtual environments ^a^	8.8 ± 3.9	8.1 ± 3.7	10.0 ± 3.9	<0.001 *
Digital skills ^a^	87.5 ± 22.6	85.1 ± 21.8	91.5 ± 23.3	0.012 *
Adaptation to university life ^a^	36.9 ± 6.5	37.0 ± 6.2	36.7 ± 7.0	0.571
Academic Satisfaction ^a^	11.2 ± 2.9	11.1 ± 2.8	11.5 ± 2.9	0.170
Pittsburgh Sleep Quality Index ^a^	8.9 ± 3.3	9 ± 3.2	8.7 ± 3.3	0.337
Sleep quality (%)				
Well	50 (16.1)	30 (60.0)	20 (40.0)	0.841
Bad	260 (83.9)	163 (62.9)	97 (37.1)	

Data expressed as mean ± SD or absolute frequency (%).

^a^ Total scores.

* p<0.05, statistically significant.

### Stepwise regression models and factors related to academic satisfaction

The generation of stepwise regression models to explain changes in academic satisfaction in medical students started from a factor pool of digital skills, adjustment to college life, emotional disturbance, and sleep quality for both males and females. The two-way elimination approach showed that for males the best model for academic satisfaction was at step 2 with the integration of adjustment to university life and depression (AIC: 544.32; RMSE: 2.42 and R2: 0.30), while for females the best model was at step 4 with the integration of adjustment to university life, depression, anxiety and sleep quality (AIC: 907.59; RMSE: 2.49 and R ^2^: 0.22) ( Table 3).

**Table 3.** Stepwise regression models of the study variables.

Variables	Addition to the model	R ^2^ adjusted	AIC	RMSE
Men				
Adaptation ^a^	Step 1	0.244	552.64	2.52
Depression	Step 2	0.302	544.32	2.42
Women				
Adaptation ^a^	Step 1	0.195	911.93	2.53
Depression	Step 2	0.165	917.93	2.58
Anxiety	Step 3	0.206	910.26	2.51
Sleep ^b^	Step 4	0.221	907.59	2.49

AIC, Akaike's information criterion; RMSE, Root mean squared error.

^a^ Adaptation to university life.

^b^ Quality of sleep.

The resulting multivariate models for the students revealed that in males increased adjustment to college life ( *x1h*) and decreased depression ( *x2h*) explained 30% of the changes in academic satisfaction *(yh*), *yh = 6.49 + 0.16*( *x1h*) - *0.15*( *x2h*). On the other hand, in females adaptation to college life ( *x1m*) and anxiety ( *x2m*) produced positive changes in academic satisfaction, unlike depression ( *x3m*) and decreased sleep quality ( *x4m*), which produced negative changes in academic satisfaction ( *ym*), *ym = 10.51 + 0.08(x1m) - 0.25(x2m) + 0.13(x3m) - 0.13(x4m*) ( Table 4).

**Table 4.** Relationship of significant factors to the academic satisfaction of medical students.

Variables	Bivariate	R ^2^	Multivariable	R ^2^
	β (95%CI)		β (95%CI)	
Men				
Adaptation ^a^	0.21 (0.14, 0.28) **	0.24	0.16 (0.09, 0.23) **	0.30
Depression	-0.24 (-0.33, -0.15) **	0.18	-0.15 (-0.24, -0.06) **	
Women				
Adaptation ^a^	0.13 (0.06, 0.19) **	0.07	0.08 (0.02, 0.14) *	0.22
Depression	-0.20 (-0.26, -0.14) **	0.16	-0.25 (-0.36, -0.14) **	
Anxiety	-0.13 (-0.33, -0.15) **	0.05	0.13 (0.01, 0.24) *	
Sleep ^b^	-0.22 (-0.34, -0.10) **	0.06	-0.13 (-0.24, -0.01) *	

β, Beta coefficient; 95% CI, 95% confidence interval.

^a^ Adjustment to university life.

^b^ Quality of sleep.

* p<0.05.

** p<0.01 statistically significant.

## Discussion

Education systems in all countries have been directly affected by the restrictions due to COVID-19, affecting about 1.57 billion students in 191 countries. ^15^ Higher education institutions were forced to migrate to virtual means in order to continue academic activities. Pre-pandemic studies showed that distance learning in medical students could lead to an increase in knowledge, yet not be effective on academic satisfaction. ^16^ Since student satisfaction is a way of addressing the quality of university services, knowing the factors that affect it has become vitally important for these institutions, especially under the remote modality in which they are currently being conducted. In this study, during the global pandemic contingency due to COVID-19, it was determined that the academic satisfaction of medical students of both sexes in the multivariate analysis was positively influenced by adequate adaptation to university life, and interestingly in women, by anxiety. On the other hand, poor sleep quality and depression had significant negative effects on academic satisfaction.

According to Huebner and Gilman, ^17^ student satisfaction is a multidimensional and complex affective variable that includes students' enjoyment and evaluation of their experiences in the educational environment. It has been verified that the difficulty of adaptation to the university environment is a factor that encourages student repetition and desertion and directly affects student satisfaction. ^18^ This study has confirmed the positive influence of adaptation to university life on academic satisfaction in both male and female medical students. Similar results were reported for first-year university students, in which it was also shown that social support networks of family and faculty members could improve academic satisfaction related to adjustment to university life. ^19^ Additionally, it has been verified that academic adjustment positively influenced the success of Netherlands university students. This success was measured in terms of their grades, number of credits earned, and intention to stay. ^20^ In Peru, university education faces a high dropout rate; according to the government regulator, it was estimated that 27% of students who enter university studies drop out during the first year. ^21^ This high dropout rate could be influenced by the difficulty of adapting to university life, which could lead to dissatisfaction and early abandonment.

Understanding and meeting students' expectations to improve their satisfaction in academic life is a challenge for universities, as students bring in multiple expectations. Efforts are needed, especially because of the potential impact of academic satisfaction on students' psychological health and well-being. ^22^ In this study it has been verified that depression negatively influences the academic satisfaction of male and female students. Depression is an important psychological problem for university students, ^23^ and the exposure of medical students to depression during their university years has been previously related to their academic satisfaction. ^24^

However, due to the pandemic context, the levels of depression in medical students have increased, mainly due to the influence of isolation, ^25^ which could generate academic problems and be reflected in their satisfaction. Universities should ensure that students have access to psychosocial services to help them cope with depression, mental distress and improve students' satisfaction with their studies.

It has been documented that the prevalence of anxiety symptoms in medical students is high. ^26^ Additionally, it has been verified that the symptom of anxiety in medical students is associated with female gender and academic performance. ^27^ Many papers show that lower academic satisfaction scores are strongly associated with psychological disorders such as anxiety, depression and stress. ^22^ Interestingly, in this study it was found that anxiety positively influences academic satisfaction in female students. In that sense, in a study with Chilean medical students it was found that those who present high levels of motivation are also more stressed and suffer greater anxiety because they want to have good results. ^24^

It has been shown that during the COVID-19 pandemic, the number of hours of sleep per night has varied among individuals, including students, which has been putting healthy lifestyles at risk. ^28^ In addition, previous studies have shown that most medical students sleep less than 6 hours per night, ^29^ and that sleep quality is a factor that directly affects academic performance and satisfaction with their studies. ^30^ In this sense, in this study more than 80% of the students had poor sleep quality, and this variable was strongly associated and negatively influenced the academic satisfaction in both males and females. University students, mainly in health sciences such as medicine, should receive more knowledge about sleep hygiene to improve satisfaction with their studies.

This study had some limitations. The majority of medical students who responded to the survey were from the first years with a predominance of women and it was conducted in a single university center. However, there was participation of students from different regions of Peru including a considerable participation of students residing abroad. Additionally, there were no cut-off scores for most of the questionnaires used, however, most of these questionnaires have been previously used in the Latino population. ^31^^,^^32^ On the other hand, we did not consider other specific factors by academic year, which could influence academic satisfaction, such as academic demand or clinical courses, because of which we could cover most common factors among medical students. However, future studies should consider these limitations and include these factors to have a better understanding of the outcome of academic satisfaction. Despite these limitations it is important to highlight the use of stepwise regression in uni- and multivariate models for the determination of the influence of each factor on academic satisfaction.

In conclusion, both sexes had a good organization and management of digital programs, the factors that influenced academic satisfaction for both sexes were adaptation to the university and depression. In addition, in women it influenced the quality of sleep and anxiety. The factors found in this study can suggest universities to implement programs to have a good mental health. In addition, this may help teachers to develop plans and strategies for adaptation to the university, in this way they could improve the academic satisfaction of medical students during their university career.

## Data availability statement

### Underlying data

Figshare: “Online academic satisfaction during the COVID-19 pandemic in medical students: role of sleep, emotions, college adjustment, and digital skills” https://doi.org/10.6084/m9.figshare.19113827.v2. ^33^

### Extended data

Figshare: “Online academic satisfaction during the COVID-19 pandemic in medical students: role of sleep, emotions, college adjustment, and digital skills”

This project contains the following extended data: https://doi.org/10.6084/m9.figshare.19119353.v4. ^34^

## Reporting guidelines

STROBE checklist for “Online academic satisfaction during the COVID-19 pandemic in medical students: role of sleep, emotions, college adjustment, and digital skills” https://doi.org/10.6084/m9.figshare.19113959. ^35^

Data are available under the terms of the Creative Commons Attribution 4.0 International license (CC-BY 4.0).

## Author’s contribution

Medina-Ramirez S.A: Conceptualization, Investigation, Software, Supervision, Visualization, Writing – Original Draft Preparation, Writing – Review & Editing; Rojas-Humpire R: Conceptualization, Investigation, Methodology, Software, Writing – Original Draft Preparation, Writing – Review & Editing; Canaza J.F: Conceptualization, Investigation, Methodology, Writing – Review & Editing; Hernandez F: Conceptualization, Investigation, Methodology, Writing – Review & Editing; Huancahuire-Vega S: Conceptualization, Investigation, Methodology, Supervision, Resources, Writing – Original Draft Preparation, Writing – Review & Editing.
