# Supplementary material for: Online academic satisfaction during the COVID-19 pandemic in medical students: role of sleep, psychological issues, college adjustment, and digital skills
Source: F1000Res. 2022 Oct 24;11:241. Originally published 2022 Feb 28. [Version 3] doi: 10.12688/f1000research.76127.3 (PMC10130700; doi:10.12688/f1000research.76127.3)
Supplement: Peer review history

# Peer review: Reviewer response for version 3

**DOI:** 10.5256/f1000research.140005.r154012

**Published:** 2023-04-25

**Authors:** Amin Nakhostin-Ansari

**Affiliations:** 1 Sports Medicine Research Center, Neuroscience Institute, Tehran University of Medical Sciences, Tehran, Iran

I would like to thank the authors for addressing all my concerns. The manuscript's quality has improved significantly after the revisions. I would suggest a language editing of the manuscript as there are issues with the manuscript's English. I have no further comments.

Is the work clearly and accurately presented and does it cite the current literature?

Partly

If applicable, is the statistical analysis and its interpretation appropriate?

Partly

Are all the source data underlying the results available to ensure full reproducibility?

Yes

Is the study design appropriate and is the work technically sound?

Partly

Are the conclusions drawn adequately supported by the results?

Partly

Are sufficient details of methods and analysis provided to allow replication by others?

No

Reviewer Expertise:

Public health, medical education, internal medicine

I confirm that I have read this submission and believe that I have an appropriate level of expertise to confirm that it is of an acceptable scientific standard.

# Peer review: Reviewer response for version 2

**DOI:** 10.5256/f1000research.134810.r145593

**Published:** 2022-08-11

**Authors:** Amin Nakhostin-Ansari

**Affiliations:** 1 Sports Medicine Research Center, Neuroscience Institute, Tehran University of Medical Sciences, Tehran, Iran

This is an interesting study evaluating the factors associated with academic satisfaction in medical students during the COVID-19 pandemic. The authors have performed a subgroup analysis in this study. They have found that different factors are related to academic satisfaction among males and females during the COVID-19 pandemic, which is one of the strengths of this study. The findings also have practical implications in the post-COVID era. However, the methods are not explained well, and there is a need to add further details to the discussion and introduction sections.

 Specific comments:

1. Depression, anxiety, and stress are evaluated in the present study. However, according to the title, the study was supposed to evaluate the role of emotion in academic satisfaction. As depression, anxiety, and stress are psychological issues and are not considered emotions, I would suggest changing the title accordingly. Also, please correct this issue throughout the manuscript.  
2. I would suggest explaining the role of sleep in medical students’ life during the COVID-19 pandemic and justifying why you evaluated it in your study.
3. Please add more details on the study setting (e.g., location and the time study was conducted) in the methods section.
4. What do you mean by “non-probability” convenience sampling?
5. Please add your exclusion and inclusion criteria for participants in the methods section.
6. I would suggest explaining the basic and demographic characteristics evaluated in your questionnaire in the methods section.
7. Please add more details on interpreting the questionnaires’ scores (e.g., what higher or lower scores mean).
8. Add more details on how PSQI’s scoring system, please.
9. You should ensure that your variables were distributed normally before using parametric tests in your study. I would suggest performing suitable tests (e.g., the Kolmogorov-Smirnov test) to determine if your variables were distributed normally. In the case of non-normal distribution, you should use non-parametric tests to compare the groups.
10. Add the response rate to the results section, please.
11. In the results section, you have categorized individuals into groups with mild to severe psychological issues. Please explain how you divided individuals into these groups and provide cut-offs in the methods section.
12. I would suggest rewriting the results of the regression analysis. Please simply mention the factors associated with academic satisfaction with their beta, 95% CI for beta, and P-values. Also, it is not obvious what are your references for independent variables. For example, do you mean depression score of DASS or having depression vs. not having depression.?
13. In the discussion section, please compare differences between males and females regarding the factors associated with academic satisfaction. Also, please compare your findings with other studies, and discuss the differences and similarities. Finally, please focus on the results of the regression analyses in your discussion. You have mentioned that sleep quality was an important factor affecting academic satisfaction in both males and females; however, sleep quality was not independently associated with academic satisfaction in males, according to your regression analysis.
14. I would suggest adding one or two sentences on the take-home message of this article to the conclusion section.

Is the work clearly and accurately presented and does it cite the current literature?

Partly

If applicable, is the statistical analysis and its interpretation appropriate?

Partly

Are all the source data underlying the results available to ensure full reproducibility?

Yes

Is the study design appropriate and is the work technically sound?

Partly

Are the conclusions drawn adequately supported by the results?

Partly

Are sufficient details of methods and analysis provided to allow replication by others?

No

Reviewer Expertise:

Public health, medical education, internal medicine

I confirm that I have read this submission and believe that I have an appropriate level of expertise to state that I do not consider it to be of an acceptable scientific standard, for reasons outlined above.

# Response

**Published:** 2022-10-18

**Authors:** Salomon Huancahuire-Vega

**Affiliations:** Peruvian Union University UPeU, Peru

1. Depression, anxiety, and stress are evaluated in the present study. However, according to the title, the study was supposed to evaluate the role of emotion in academic satisfaction. As depression, anxiety, and stress are psychological issues and are not considered emotions, I would suggest changing the title accordingly. Also, please correct this issue throughout the manuscript.

 Reply. Thank you for this suggestion. We have followed the recommendation and change the title, we have also revised and corrected the entire article:

 “Online academic satisfaction during the COVID-19 pandemic in medical students: role of sleep, psychological issues, college adjustment, and digital skills”

 2. I would suggest explaining the role of sleep in medical students’ life during the COVID-19 pandemic and justifying why you evaluated it in your study.

 Reply. Thank you for this suggestion. We have added information on the role of sleep in medical students during the pandemic in the introduction section:

 “Likewise, it has been observed that the interruption of face-to-face daily activities, social isolation could affect healthy sleep, altering the times of going to bed and waking up, the appearance of sleep disorders, and promoting the development of psychological issues (stress, depression, anxiety).”

 3. Please add more details on the study setting (e.g., location and the time study was conducted) in the methods section.

 Reply: Thank you for your suggestion. We have added more information about the study:

 “We conducted the study from last week of January, to first week of March, 2021, a virtual meeting was held for students of each academic year and each week a reminder to complete the survey was sent.”

 4. What do you mean by “non-probability” convenience sampling?

 Reply. Thank you for this question. We use "non-probability" because convenience sampling is a non-random, non-probability sampling technique. We recruited medical students who had access to a mobile device with internet connection and had time to complete the survey. We now this in the article:

 “We used convenience sampling to enlist the students, who were invited to complete an online survey, in which questionnaires related to academic satisfaction, adaptation to the university, digital skills, sleep quality, stress, depression and anxiety were applied.”

 5. Please add your exclusion and inclusion criteria for participants in the methods section.

 Reply. Thank you for your comment. We have added information about inclusion/exclusion criteria in the methods section:

 “Inclusion criteria were: 1) Students enrolled during the 2021 academic cycle, 2) who have not completed another health career. Students who didn't give their informed consent or didn't complete the survey were excluded from the study.”

 6. I would suggest explaining the basic and demographic characteristics evaluated in your questionnaire in the methods section.

 Reply. Thank you for your comment. We have added more information about socio demographic characteristics in the methods section:

 “Socio-demographic aspects were considered, such as year of study, country of origin, place of residence, in order to know the general information of the participants…”

 7. Please add more details on interpreting the questionnaires’ scores (e.g., what higher or lower scores mean).

 Reply. Thank you for your recommendation: Information on the interpretation of each questionnaire’s score has been added.

 8. Add more details on how PSQI’s scoring system, please.

 Reply. Thanks for your comment. We have added more details about PSQI’s scoring system in the methods section:

 “The questionnaire consists of 9 self-assessment questions, 4 subjective (questions 1-4) and 5 objective (questions 5-9) questions, that are used to obtain the global score. These questions are distributed in 7 components, which are: subjective sleep quality, latency, duration, efficiency, sleep disturbances, use of medication for sleep, and daytime dysfunction. Each component, have instructions to sum the score of the subjective and objective questions. High scores (Global score >5) indicate poor sleep quality.”

 9. You should ensure that your variables were distributed normally before using parametric tests in your study. I would suggest performing suitable tests (e.g., the Kolmogorov-Smirnov test) to determine if your variables were distributed normally. In the case of non-normal distribution, you should use non-parametric tests to compare the groups.

 Reply. Thanks for your comment. In the exploratory analysis we used the Kolmogorov-Smirnov test, then we use a non-parametric test (U de Mann Whitney) for comparative analysis. We have added this specification in the data analysis section:

 “Data analysis was performed in the R programming language version 4.0.2. (https://www.rstudio.com/) (RRID:SCR_001905). The variables were ordered in graphs and tables taking into account their categorical nature expressed as absolute frequency and percentage (%), or numerical as mean ± standard deviation (SD). For the comparative analysis, the χ 2 (chi-square) or Mann Whitney U test was used according to the non-normal distribution of the variable evaluated by the Kolmogorov-Smirnov test. To establish the best model of factors related to academic satisfaction in medical students, stepwise regression models were performed with bidirectional elimination approach in the adjustment of independent variables. The best regression models were established based on Akaike's information criterion (AIC), root mean square error (RMSE), and R ^2^. Multivariate regression models stratified by sex were obtained with their respective 95% confidence intervals (95%CI). A value of p<0.05 was considered statistically significant in the analyses.

 10. Add the response rate to the results section, please.

 Reply. Thank you for your comment. We have added the response rate in the results section:

 “A total of 310 responses were obtained (rate of responses of 70%).”

 11. In the results section, you have categorized individuals into groups with mild to severe psychological issues. Please explain how you divided individuals into these groups and provide cut-offs in the methods section.

 Reply. Thank you for your comment, you are right. We have added the cut-offs of stress, depression, and anxiety in the method section:

 “According to the score obtained in the specific psychological issue it is classified as mild (anxiety: 4, stress: 8-9, depression: 5-6), moderate (anxiety: 5-7, stress: 10-12, depression: 7-10), severe (anxiety: 8-9, stress: 13-16, depression: 11-13) and extremely severe (anxiety ≥ 10, stress ≥ 17, depression ≥ 14). In its Spanish version it has an acceptable internal validity, for stress (α=0.82), depression (α=0.84) and anxiety (α=0.70). and shows a good interrelation between the 3 factors evaluated by this scale. ^15^”

 12. I would suggest rewriting the results of the regression analysis. Please simply mention the factors associated with academic satisfaction with their beta, 95% CI for beta, and P-values. Also, it is not obvious what are your references for independent variables. For example, do you mean depression score of DASS or having depression vs. not having depression.?

 Reply. Thank you for your suggestion. We have rewrite the section “Stepwise regression models and factors related to academic satisfaction”:

 “Multivariate models in males showed that a higher score in adaptation (β: 0.16, 95% CI 0.09 to 0.23, p<0.01) and a lower score of depression (β: -0.15, 95% CI -0.24 to -0.06, p<0.01) were related to increased student satisfaction and explain 30% of the changes in the adjusted model. On the other hand, in females a higher score of adaptation (β: 0.08, CI 95% 0.02 to 0.14, p<0.01) and anxiety (β: 0.13, CI95% 0.01 to 0.24, p<0.01), while, a lower score in depression (β: -0. 25, 95%CI -0.36 to -0.14, p<0.01) and sleep quality (β: -0.13, 95%CI -0.24 to -0.01, p<0.01) were related to academic satisfaction. Overall, the model for women explained that 22% of changes in academic satisfaction.”

 13. In the discusión section, please compare differences between males and females regarding the factors associated with academic satisfaction. Also, please compare your findings with other studies, and discuss the differences and similarities. Finally, please focus on the results of the regression analyses in your discussion. You have mentioned that sleep quality was an important factor affecting academic satisfaction in both males and females; however, sleep quality was not independently associated with academic satisfaction in males, according to your regression análisis.

 Reply. Thank you for your comment. We have focused the discussion on the regression analysis and we have added comments about differences between males and females regarding to anxiety and sleep quality. By the other hand, you are right about sleep quality as factor affecting academic satisfaction only in females. We have corrected the discussion this aspect:

 “For example, a study conducted among medical students showed a significant gender difference with twice the rate of anxiety among women compared to men (SECOND NEW REFERENCE ADDED). Another study shows that the highest rates of anxiety in female students occur in the first years of study (THIRD NEW REFERENCE ADDED). To explain the difference in anxiety levels between male and female students, it has been verified that female Chinese university students had higher levels of anxiety than males and these anxiety levels were associated with their body image, alcohol consumption habits and academic performance (FOURTH NEW REFERENCE ADDED).”

 “Additionally, one study highlighted the top three student concerns associated with anxiety: academic performance, pressure to succeed, and post-graduation plans (FIFTH NEW REFERENCE ADDED). More studies are required to determine the factors related to mental problems such as anxiety that influence the academic achievement of medical students.”

 “During the COVID-19 pandemic, it has been verified that compared to men, women reported lower quality and efficiency of sleep and greater symptoms of insomnia (SIXTH NEW REFERENCE ADDED). This difference is probably due to women being more concerned about their family's health and the risk of exposure to the coronavirus (SEVENTH NEW REFERENCE ADDED). The gender difference seems to play a role in influencing sleep quality and satisfaction with studies in medicine students. This difference should be taken into account when planning educational interventions for medical students on the importance of proper sleep hygiene and the consequences of poor sleep practices. Additionally, university students, mainly in health sciences such as medicine, should receive more knowledge about sleep hygiene to improve satisfaction with their studies.”

 “On the other hand, in both males and females, depression had significant negative effects on academic satisfaction and in relation to poor sleep quality, interestingly, it only affected females.”

 “In this sense, in this study more than 80% of the students had poor sleep quality, and this variable was strongly associated and negatively influenced the academic satisfaction in female students.”

 14. I would suggest adding one or two sentences on the take-home message of this article to the conclusion section.

 Reply. Thank you for your comment. We have added take-home messages in the conclusion section:

 “The factors found in this study can suggest universities to implement programs to have a good mental health and to educate medical students about the importance of proper sleep hygiene. In addition, this may help teachers to develop plans and strategies for adaptation to the university, in this way they could improve the academic satisfaction of medical students during their university career.”

# Peer review: Reviewer response for version 1

**DOI:** 10.5256/f1000research.80084.r127163

**Published:** 2022-04-26

**Authors:** Oriana Rivera Lozada

**Affiliations:** 1 South American Center for Education and Research in Public Health, Universidad Norbert Wiener, Lima, Peru

The article addresses a very interesting topic for medical education today, such as the topic of satisfaction in times of Covid. The document is quite clear and concrete. However, it could be improved in some aspects:

 Introduction:

 It is well structured, however, it can be strengthened with information from Peruvian studies that have been carried out (background).

 Methodology:

1. Add the reliability data of the instrument Adaptability to university life.
2. Specify sample and sampling data

 Results and Discussion:

 Add the biases of the study and what they did to control them. The discussion should strengthen with preferably national background.

 Specify some interventions that can be proposed based on this research.

Is the work clearly and accurately presented and does it cite the current literature?

Yes

If applicable, is the statistical analysis and its interpretation appropriate?

Yes

Are all the source data underlying the results available to ensure full reproducibility?

Yes

Is the study design appropriate and is the work technically sound?

Yes

Are the conclusions drawn adequately supported by the results?

Yes

Are sufficient details of methods and analysis provided to allow replication by others?

Yes

Reviewer Expertise:

Public Health, epidemiology, infectious diseases and health education.

I confirm that I have read this submission and believe that I have an appropriate level of expertise to confirm that it is of an acceptable scientific standard.

# Response

**Published:** 2022-06-06

**Authors:** Salomon Huancahuire-Vega

**Affiliations:** Peruvian Union University UPeU, Peru

*Introduction:*

*It is well structured, however, it can be strengthened with information from Peruvian studies that have been carried out (background).*

**Thank you for your comment. We have added information about Peruvian studies:**

**“In addition, their clinical practices were suspended as a protective measure. Under these conditions, it was determined in Peruvian health students that they had low levels of knowledge, risk perception attitudes, and preventive practices regarding COVID-19”**

*Methodology:*

*Add the reliability data of the instrument Adaptability to university life.*

**Thank you for your comment. We have added reliability information about Adaptability to university life instrument:**

**“Regarding its reliability evaluated by the test-retest procedure, a correlation of 0.821 was recorded, which implies a good reliability (greater than 0.8)”**

*Specify sample and sampling data*

**Thank you for your comment. We have added information about sampling data**

**“Participants were selected by non-probability convenience sampling”**

*Results and Discussion:*

*Add the biases of the study and what they did to control them. The discussion should strengthen with preferably national background.*

**Thank you for your comment. In the last part of the discussion we put a paragraph with the limitations and possible biases of the study, we also commented on explanations for each of them.**

**“This study had some limitations. The majority of medical students who responded to the survey were from the first years with a predominance of women and it was conducted in a single university center. However, there was participation of students from different regions of Peru including a considerable participation of students residing abroad. Additionally, there were no cut-off scores for most of the questionnaires used, however, most of these questionnaires have been previously used in the Latino population. ^^On the other hand, we did not consider other specific factors by academic year, which could influence academic satisfaction, such as academic demand or clinical courses, because of which we could cover most common factors among medical students. However, future studies should consider these limitations and include these factors to have a better understanding of the outcome of academic satisfaction. Despite these limitations it is important to highlight the use of stepwise regression in uni- and multivariate models for the determination of the influence of each factor on academic satisfaction”**

*Specify some interventions that can be proposed based on this research.*

**Thank you for your comment. Given that depression and anxiety influenced academic satisfaction, it is proposed that universities implement programs to monitor the mental health of students. These recommendations are at the end of the conclusion paragraph:**

**“The factors found in this study can suggest universities to implement programs to have a good mental health. In addition, this may help teachers to develop plans and strategies for adaptation to the university, in this way they could improve the academic satisfaction of medical students during their university career”**
